# Fibrocartilaginous Dysplasia of the Bone: A Rare Variant of Fibrous Dysplasia

**DOI:** 10.7759/cureus.448

**Published:** 2016-01-05

**Authors:** Raju Vaishya, Amit Kumar Agarwal, Nishint Gupta, Vipul Vijay

**Affiliations:** 1 Orthopaedics, Indraprastha Apollo Hospitals; 2 Orthopaedics, St. Stephens hospital

**Keywords:** fibrous dysplasia, fibrocartilaginous dysplasia

## Abstract

Fibrocartilaginous dysplasia (FCD) is a rare variant of fibrous dysplasia (FD) which frequently involves the long bones, and the proximal femur is the most commonly affected site. This benign, lytic, and expansile bone lesion causes progressive deformity in the bones and may lead to pathological fracture. Radiologically, this lesion may mimic cartilaginous benign and malignant bone tumors. Therefore, histopathological differentiation of FCD from other cartilaginous tumors is of the utmost importance. The treatment is often surgical, in the form of curettage and bone grafting or corrective osteotomy, to treat progressive deformity in the long bones. The risk of pathological fracture is high in FCD with bony deformity and often requires surgery.

## Introduction

Fibrous dysplasia (FD) is a benign fibro-osseous lesion that occurs due to the developmental anomaly of the bone [1]. It may exist as the monostotic, polyostotic, or craniofacial form. Histologically, it is characterized by the spindle cell embedded in fibrous stroma along with irregularly shaped trabeculae of immature bone in place of normal bone and marrow [2]. The FD has a predilection for long bones, skull, ribs, and jaw [3]. Radiologically, FD is usually a well-defined intramedullary and expansile lesion. It may show variation in the density ranging from radiolucent to dense, depending on the relative proportions of the fibrous and osseous tissue [4].

Infrequently, the foci of cartilaginous tissue may be present in a polyostotic or monostotic type of FD. This rare variation of FD may also show extensive cartilaginous tissue within the fibro-osseous tissue. This rare variation in FD has been labeled as “Fibrocartilaginous Dysplasia” in the literature [5]. A fibrocartilaginous dysplasia (FCD) commonly occurs in the lower extremities, especially in the proximal femur, leading to disabling deformity of the limb [6]. Awareness about this rare variant of FD is necessary to reach an accurate diagnosis. Sometimes, it may be confused as chondrosarcoma arising from FD or other benign or malignant cartilaginous tumor of the bone [7]. We share our experience in the management of a case of a 17-year-old male suffering with FCD in the right proximal femur with a classical ‘shepherd crook’ deformity.

## Case presentation

A 17-year-old male presented with a painful limp and a history of a progressive deformity of the right hip over the last four years. A curettage and bone grafting was done of the right proximal femur two years ago, and the histopathological report was suggestive of FD. He got relief from the symptoms for only one year, but his symptoms recurred in the form of pain, stiffness, shortening, and limp. On examination, there was a large globular, tender swelling (10 x 8 x 4 cms) around the right hip and proximal thigh. There was a healed surgical scar on the lateral aspect of the thigh, and the range of motion of the hip was reduced, with a 6 cm shortening of the affected limb. The distal neurovascular status of the right lower limb was within normal limits.

**Figure 1:**
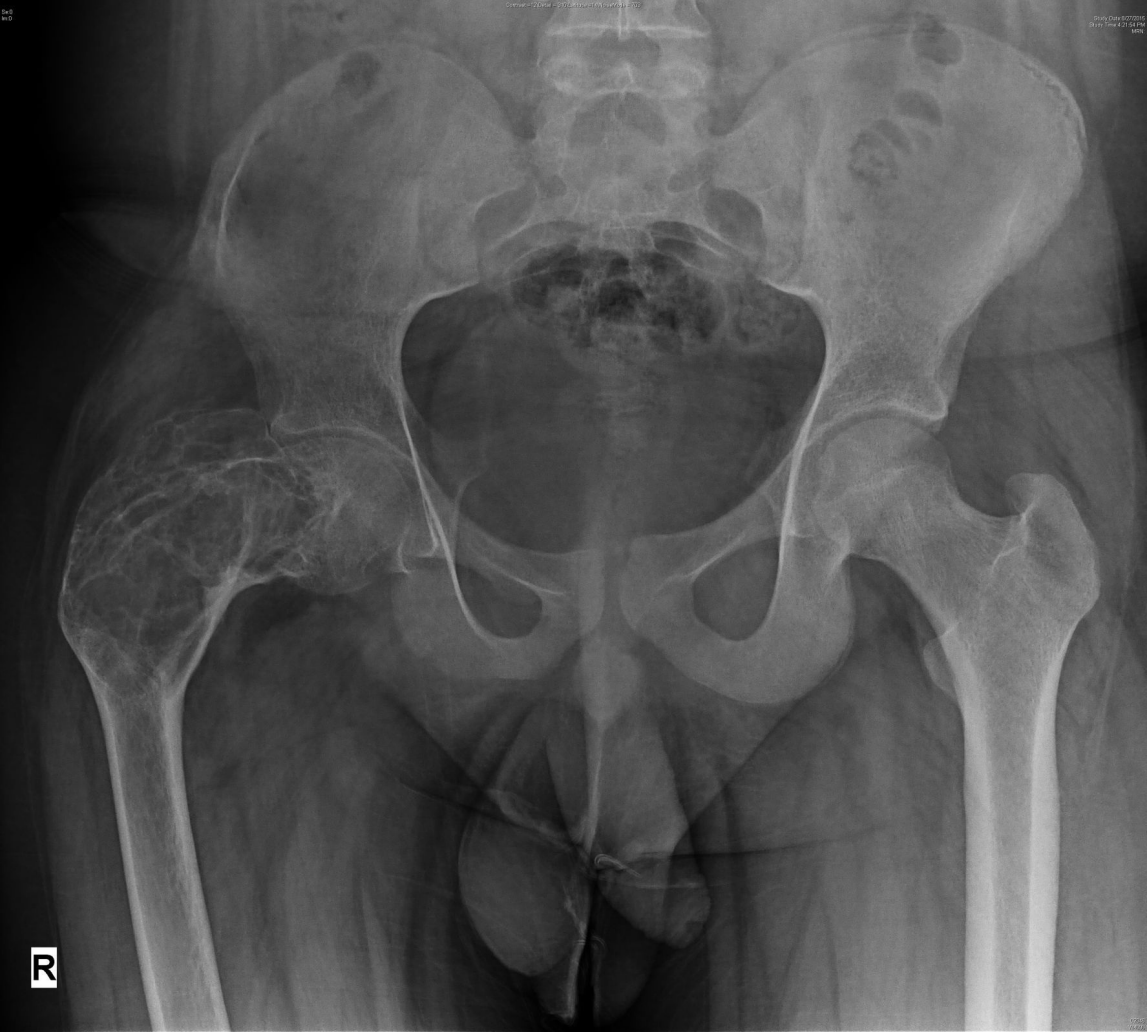
Anteroposterior X-rays of the pelvis with both hips showing large, well-defined, expansile lytic lesion in the metaphysis of the proximal femur with a breach in the lateral cortex

Anteroposterior (AP) and lateral view radiographs of the hip and proximal thigh were done (Figure 1), which revealed a large, well-defined, expansile lytic lesion in the metaphysis of the proximal femur extending up to the subtrochanteric region of the femur. Both the cortices in the metaphyseal region were thinned out, with a breach in the lateral cortex. The neck shaft angle was reduced to 84 degrees, resulting in a severe coxa vara deformity, along with anterolateral bowing of the proximal femur resembling the "shepherd's crook deformity" of FD [8].

**Figure 2:**
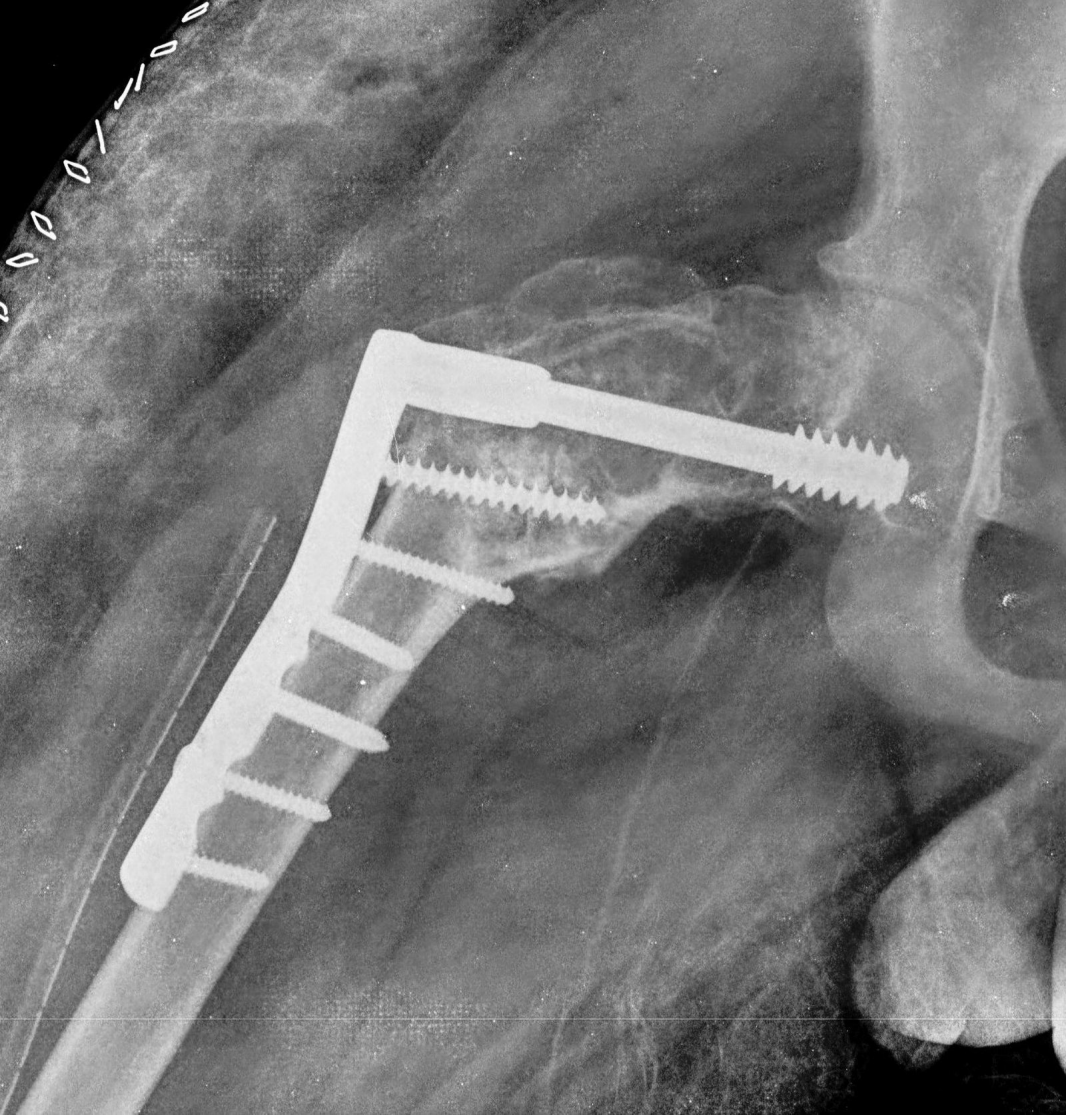
Anteroposterior X-rays of the hip with thigh, immediately post op showing a valgus osteotomy at the subtrochanteric region and fixed with a 95 degree Dynamic condylar screw (DCS) and barrel plate

Considering his current problems, it was decided to do extensive curettage, bone grafting, and corrective osteotomy with internal fixation. Informed patient consent was obtained. Surgery was performed on a fracture table through a lateral incision and a plane was dissected between gluteus medius and tensor fascia lata muscle. The lesion was curetted extensively; a valgus osteotomy was done at the subtrochanteric region, keeping the laterally closed wedge of 3 cm. The osteotomy was fixed with a 95-degree dynamic condylar screw (DCS) and a plate (Figure 2).

**Figure 3:**
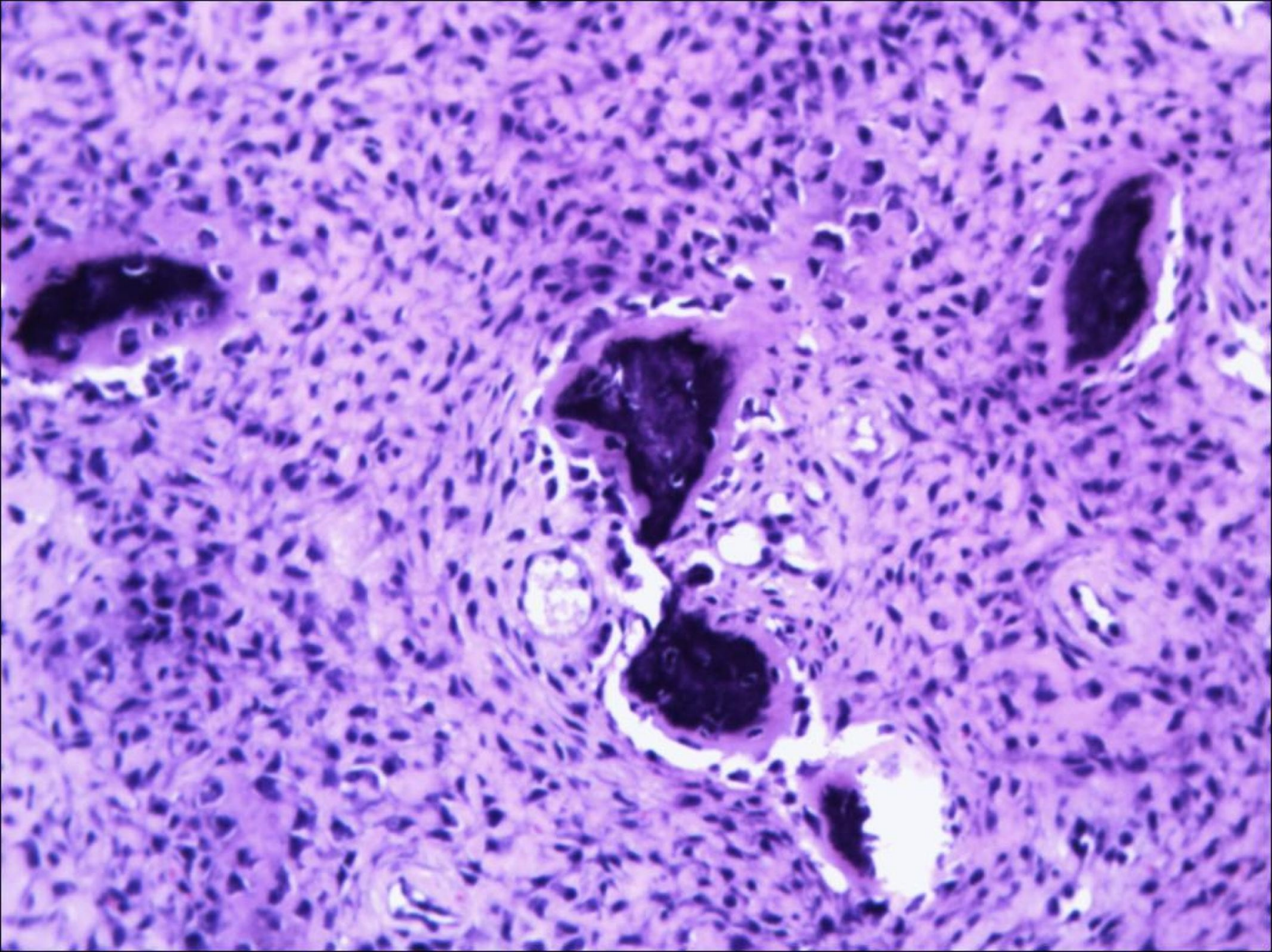
Histopathological examination (HPE) revealed spindle to stellate stromal cells embedded in dense fibrous stroma

The bony cavity was filled with allografts. The removed tissue was fibrocartilaginous in nature, which, on histopathological examination (HPE), revealed spindle to stellate stromal cells embedded in the dense fibrous stroma, along with dense collagen matrix surrounded by thin-walled blood vessels and a few widely scattered thin rims of woven bone, mostly devoid of osteoblastic rimming (Figure 3).

**Figure 4:**
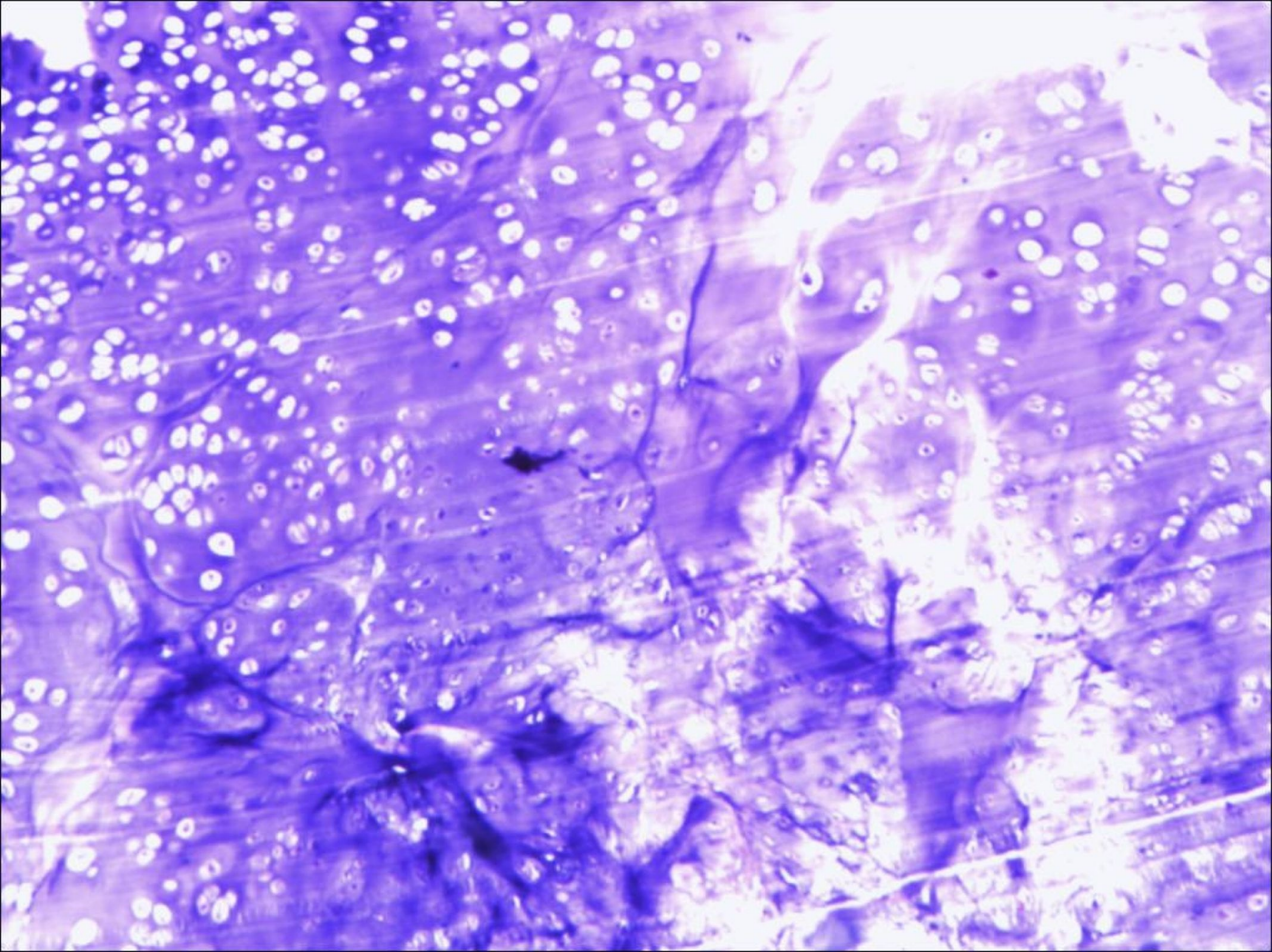
The tissue showed features of a benign mesenchymal lesion with bone matrix and chondroid matrix formation consistent with the characteristics of FCD

The tissue showed features of a benign mesenchymal lesion with bone matrix and chondroid matrix formation consistent with the characteristics of FCD (Figure 4).

**Figure 5:**
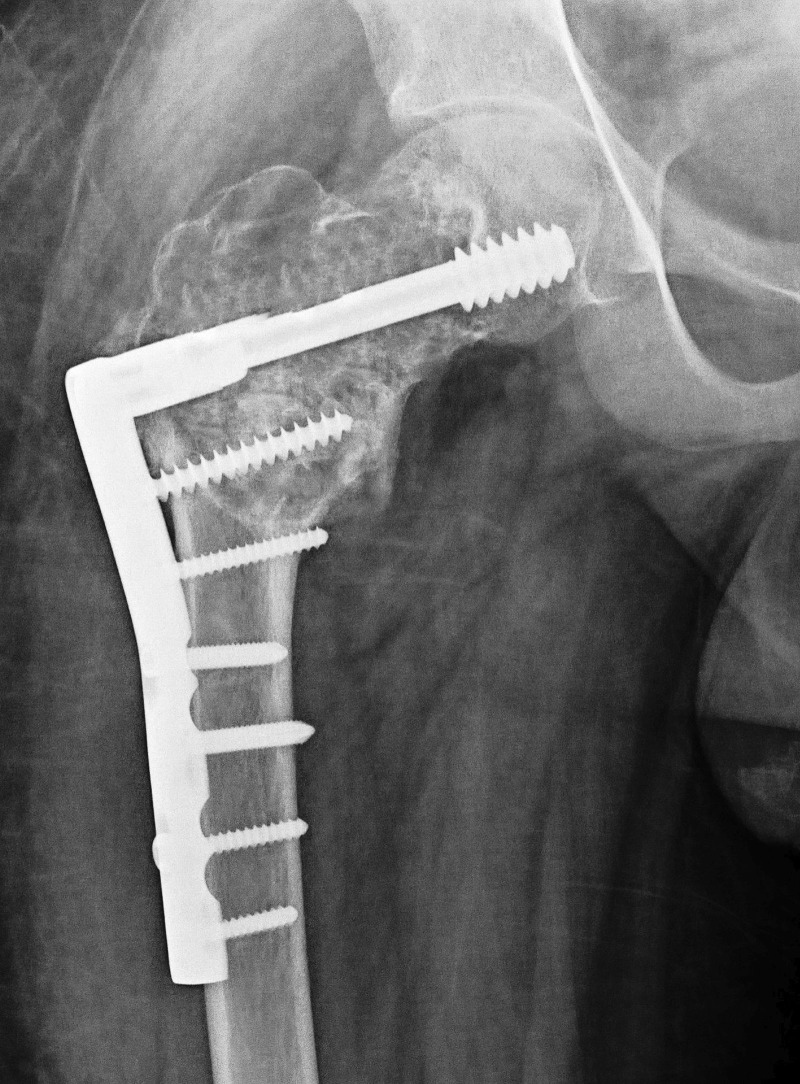
Anteroposterior X-rays of the hip with thigh at the one-year follow-up showing correction of the varus and improvement in the lytic lesion of the bone

On the second postoperative day, non-weight bearing mobilization was allowed with the help of a walker. At the end of one year, the patient was doing well, and the shortening and limp have improved significantly with the radiological union of the osteotomy; there have been no signs of recurrence of the tumor or deformity (Figure 5).

## Discussion

The FCD is a rarer variety of FD, which shows the presence of cartilaginous tissue in variable amount along with fibro-osseous tissue. Pelzmann, et al. first reported FCD as a variant of FD in a 20-year-old man with polyostotic fibrous dysplasia [9].

Various conditions may mimic FCD radiologically. These include enchondroma, chondrosarcoma, and fibrocartilaginous mesenchymoma. These conditions can be differentiated with FCD by histopathology, site of involvement, and age group (Table 1).

**Table 1:** Comparison of Differentials of Cartilaginous Bone Tumors Resembling FCD

Bone Tumors	Age Group	Clinical Presentation	Histological Appearance	Radiological Features
Enchondroma	10-30 years	Mostly in hands and feet Asymptomatic Pathological fracture Malignant transformation (low-grade chondrosarcoma)	Hyaline cartilage Myxoid degeneration Endochondral ossification Calcification	Lytic lesion Sharply defined scalloped margins Ring and arc calcification No periosteal reaction
Chondrosarcoma	< 5% in firstand second decades	Focal pain Association with Ollier’s disease Maffucci’s syndrome Hereditary multiple exostosis	Mesenchymal Clear cell Myxoid varieties Lack of fibro-osseous tissue	Expansile Sclerotic and centrally lucent Narrow transition zone Ring and arc calcification
Fibrocartilaginous Mesenchymoma	Rare in first and second decades	Affects long bones Locally aggressive tumor Rapid increase in size	Unique epiphyseal plate-like cartilage Fibrous stroma	Osteolytic lesion Expansile
Fibrocartilaginous Dysplasia	Common in first and second decades	Common in lower extremities (proximal femur) Progressive deformity Risk for pathological fracture	Cartilage lobules Fibro-osseous tissue	Osteolytic Expansile

Enchondroma is a common benign cartilaginous tumor that occurs mostly in the small tubular bones of the hands and feet. Histologically, it consists of mature lobules of hyaline cartilage with foci of myxoid degeneration, calcification, and endochondral ossification. Chondrosarcoma is distinctly uncommon in childhood. It comprises 5% or fewer of all primary malignant skeletal tumors in the first two decades of life. However, chondrosarcoma may show an association with enchondromatosis syndromes, e.g. Ollier`s disease, Maffucci`s syndrome, metachondromatosis, and other conditions like hereditary multiple exostosis that usually presents in the first two decades of life. In children, three variants of chondrosarcoma are recognized: mesenchymal, clear cell, and myxoid. Histologically, these variants of chondrosarcoma lack the fibro-osseous component characteristic of fibrous dysplasia. Fibrocartilaginous mesenchymoma is an extremely rare lesion of the bone in children and adolescents that affects the metaphyseal region of long bones, particularly the proximal fibula. The lesion contains both fibrous and cartilaginous tissue that shows a pattern of the unique epiphyseal plate-like cartilage with dense fibrous stroma, along with the gross destruction of the surrounding cortical bone. It is locally aggressive with a high recurrence rate, especially when resection is incomplete.

The histological appearance of FCD consists of large lobules of cartilage surrounded by fibro-osseous tissue with features typical of fibrous dysplasia. The amount of cartilaginous component may vary. These large dysplastic cartilaginous islands may be misinterpreted as benign or even malignant cartilaginous tumors.

The frequent involvement of the proximal femur, along with the progressive deformity, has been mentioned in few reported cases of FCD. Similarly, in this case, there was a gradually progressive deformity in the right hip as the lesion was in the proximal femur and involved the greater trochanter, thus, causing dysfunction of the muscles around the hip, including the abductor and flexor group of muscles. The range of motion was grossly reduced in all directions. The limping was due to a 6 cm shortening, along with the antalgic component. Also, there was severe, progressive coxa vara in the hip as well as excessive thinning of both the lateral and medial cortex, leading to the risk of pathological fracture. The classic shepherd's crook deformity is a severe form of coxa vara where there is a reduction in the neck shaft angle beyond 90 degrees, as in this case [10]. The shape of the proximal femur resembles that of the staff carried by herders (shepherds), which is known as a crook. The most common cause of this deformity is fibrous dysplasia, but it could also be due to other conditions like Paget’s disease, osteogenesis imperfecta, congenital or developmental coxa vara, Perthes disease, sequelae of osteomyelitis of the proximal femur, malunited proximal femoral fracture, cleidocranial dysostosis, and Vitamin D deficiency (rickets and osteomalacia) (Table 2).

**Table 2:** Differential Diagnosis of Shepherd's Crook Deformity of the Proximal Femur

S.No.	Cause	Differential Diagnosis
1	Congenital	Coxa vara Osteogenesis imperfecta Cleidocranial dysostosis
2	Developmental	Perthes disease (sequelae) Coxa vara
3	Tumor	Fibrous dysplasia Fibrocartilaginous dysplasia
4	Metabolic	Rickets Osteomalacia Pagets disease
5	Infection	Chronic osteomyelitis (sequelae)
6	Trauma	Malunited proximal femur fractures

The correction of the coxa vara deformity and the shortening required a valgus osteotomy and filling of the bony cavity with bone allograft. The extensive curettage of the lesion, correction of the deformity, and a stable fixation allowed the better outcome of the disease and early rehabilitation in this patient. 

## Conclusions

FCD is a rare bone lesion in the proximal femur that needs to be surgically treated early as it causes progressive deformity and may predispose the bone for pathological fracture. The diagnosis of this rare entity may be misleading radiologically and requires histopathological confirmation. We recommend an extensive curettage, bone grafting, and correction of the deformity with an osteotomy and internal fixation. Although FCD has been described as a histological variant of FD, no clinical difference has been found between the two entities.
